# Australian health care providers’ views on opt-out HIV testing

**DOI:** 10.1186/s12889-015-2229-9

**Published:** 2015-09-14

**Authors:** Stacy Leidel, Ruth McConigley, Duncan Boldy, Sally Wilson, Sonya Girdler

**Affiliations:** School of Nursing, Midwifery and Paramedicine, Faculty of Health Sciences, Curtin University, Perth, WA Australia; School of Occupational Therapy and Social Work, Faculty of Health Sciences, Curtin University, Perth, WA Australia

## Abstract

**Background:**

Opt-out HIV testing is a novel concept in Australia. In the opt-out approach, health care providers (HCPs) routinely test patients for HIV unless they explicitly decline or defer. Opt-out HIV testing is only performed with the patients’ consent, but pre-test counselling is abbreviated. Australian national testing guidelines do not currently recommend opt-out HIV testing for the general population. Non-traditional approaches to HIV testing (such as opt-out) could identify HIV infections and facilitate earlier treatment, which is particularly important now that HIV is a chronic, manageable disease. Our aim was to explore HCPs’ attitudes toward opt-out HIV testing in an Australian context, to further understanding of its acceptability and feasibility.

**Methods:**

In this qualitative study, we used purposeful sampling to recruit HCPs who were likely to have experience with HIV testing in Western Australia. We interviewed them using a semi-structured guide and used content analysis as per Graneheim to code the data. Codes were then merged into subcategories and finally themes that unified the underlying concepts. We refined these themes through discussion among the research team.

**Results:**

Twenty four HCPs participated. Eleven participants had a questioning attitude toward opt-out HIV testing, while eleven favoured the approach. The remaining two participants had more nuanced perspectives that incorporated some characteristics of the questioning and favouring attitudes. Participants’ views about opt-out HIV testing largely fell into two contrasting themes: normalisation and routinisation versus exceptionalism; and a need for proof versus openness to new approaches.

**Conclusion:**

Most HCPs in this study had dichotomous attitudes toward opt-out HIV testing, reflecting contrasting analytical styles. While some HCPs viewed it favourably, with the perceived benefits outweighing the perceived costs, others preferred to have evidence of efficacy and cost-effectiveness.

**Electronic supplementary material:**

The online version of this article (doi:10.1186/s12889-015-2229-9) contains supplementary material, which is available to authorized users.

## Background

Opt-out HIV testing is a novel concept in Australia. In the opt-out approach, health care providers (HCPs) routinely test patients for HIV unless they explicitly decline or defer. Opt-out HIV testing is only performed with the patients’ consent, but pre-test counselling is abbreviated [1]. This approach to HIV testing uses the behavioural economics concept of default bias, which is the propensity to choose inaction over action [2, 3] Australian national testing guidelines do not currently recommend opt-out HIV testing for the general population. HIV testing in Australia is “opt-in”, which means that testing is conducted according to risk factors (such as sexual or drug use practices), clinical indication (such as having another sexually transmitted infection), or by patient request [1]. However, there has been a recent increase in HIV infections in Australia, with the highest number of HIV diagnoses in 20 years recorded in 2013 [4]. In addition, up to 50 % of patients diagnosed with HIV have already developed immune deficiency [4]. An Australian study showed that over half of the people with HIV sought health care in the year prior to their diagnosis [5] which indicates that opportunities to test patients for HIV are being missed. Non-traditional approaches to HIV testing (such as opt-out) could identify HIV infections and facilitate earlier treatment, which is particularly important now that HIV is a chronic, manageable disease [6, 7]. Opt-out testing approaches to other sexually transmitted infections like chlamydia, gonorrhoea, and hepatitis B have been found to be cost-effective and acceptable to patients [8–10]. Experimental research has shown that opt-out testing is particularly effective in increasing testing rates and patient acceptance of stigmatised diseases such as HIV [11].

Results published in systematic reviews have shown that opt-out HIV testing is generally acceptable to HCPs [12–14]. However, these reviews were conducted in settings that may not be generalizable to Australia, such as low-and middle-income countries [12] or sub-Saharan Africa [13]. One systematic review of HIV testing in resource-rich countries included three Australian studies, but these studies did not address the opt-out approach or testing in the general population [14]. A US systematic review addressed operational aspects of opt-out HIV testing, which may not be transferable to the Australian context due to differences between the countries’ health systems [15]. A review of opt-out HIV testing in Australian antenatal clinics suggested that it was effective (expectant mothers are the only group in which opt-out HIV testing is recommended in Australia), but it may not be applicable to the general Australian population [16]. Despite the plethora of international research on the topic, it is not known to what extent opt-out HIV testing is appropriate, acceptable, or feasible to HCPs in general health care settings in Australia. Two Australian studies indicated that educational barriers (such as a lack of HIV content during training) and operational barriers (such as time constraints) prevent Australian HCPs from seeing HIV as relevant to their practice. Informants also stated that Australian general practitioners should make HIV testing a routine part of their practice [17, 18].

Acceptability and feasibility of the opt-out approach to HIV testing should be thoroughly explored from an HCP perspective before considering a change in testing practice. Diagnostic testing differs from other health interventions because only physicians (or other qualified HCPs) are legally permitted to order and receive financial reimbursement for tests. Fundamentally, every HIV test is the end result of an HCP’s decision-making process, which is affected by his or her attitudes, knowledge, experience, and training; and is influenced by colleagues, professional organisations, health systems, and financial incentives. This means that a change in HIV testing practice cannot occur without exploring the attitudes that influence HCPs’ decision-making processes. Our aim was to explore HCPs’ attitudes toward opt-out HIV testing in an Australian context, to further understanding of its acceptability and feasibility.

## Method

### Participants

We conducted this qualitative research in Western Australia. Using purposeful and snowball sampling, we recruited participants who were likely to have experience with HIV testing [19]. The sample included primary care nurse practitioners (NPs), general practitioners (GPs), and physician specialists in relevant fields (such as public health) who perform HIV testing in their practice. Among these participants, we chose a variety of ages, settings, and years in practice, to enhance data richness and diversity. We estimated that 20–25 participants would be required to reach data saturation. Between April and November 2014, one researcher (SL) conducted the interviews using a semi-structured guide [see Additional file 1]. Sampling continued until data saturation was reached. This study was approved by the Human Research Ethics Committee at Curtin University. All participants gave written consent to be interviewed.

### Data analysis

The interviews were audio-recorded and transcribed verbatim. After transcription we read each transcript several times for data immersion. Using content analysis as described by Graneheim, one researcher (SL) coded the transcripts, which involved placing words or segments of text into categories based on consistency of meaning [20]. Next, the transcripts were independently coded by an experienced qualitative researcher (RM) to enhance the codes’ reliability. We then refined the codes through discussion and frequent review of the raw data to find supporting evidence for each code, which was an iterative process that took place over several months [21]. We then combined the codes into subcategories based on similarity of meaning, which we then merged into themes that unified the underlying concepts. We refined these themes through discussion among the research team, and conducted four member checks to confirm theme validity and enhance rigor [22]. We used NVivo software to organise the qualitative data.

## Results

We conducted 24 semi-structured interviews. The age of the participants ranged from 31 to 66, with a mean age of 43 years (median = 43; SD = 8.7). Forty-one percent (N = 10) of the participants were female. The mean number of hours per week the participants spent in clinical practice was 28.7 (median = 32.5; SD = 14.8). Seventeen participants were GPs, five were NPs, and two were physician specialists. The number of years since completing general practice or specialty training (or masters’ level training for NPs) ranged from one to 43, with a mean of 13.5 years (median = 10, SD = 11.3). Whilst participants were often unsure, the number of estimated HIV tests they had ordered in the previous year ranged from zero to 1000. Similarly, the number of HIV-positive diagnoses participants estimated to have made over the course of their career ranged from zero to 100. (See Table 1).

**Table 1:** Participant characteristics

Participant characteristic	N (%)	Range
Male	14 (58.3)	
Profession		
GP	17 (70.8)	
NP	5 (20.8)	
Specialty physician	2 (8.3)	
	Mean (SD)	
Age (years)	43.8 (8.7)	31–66
	Median	
Years since specialty training completion	10	1–43
Hours worked per week	32.5	0–42.5
Estimate of HIV tests performed in preceding year	49	0–1000
Estimate of HIV-positive diagnoses during career	N (%)	
Nil	8 (33.2)	
1–5	10 (41.5)	
6–10	2 (8.3)	
>10	4 (16.6)	

**Fig. 1:**
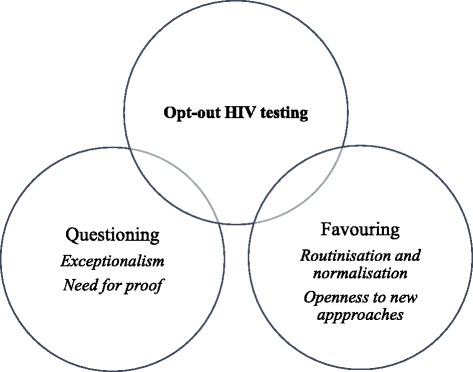
Concept map of themes

We present two findings that emerged from the data. Participants’ views about opt-out HIV testing largely fell into two contrasting themes: normalisation and routinisation versus exceptionalism; and a need for proof versus openness to new approaches. Eleven participants had a questioning attitude toward opt-out HIV testing, while eleven favoured the approach. The remaining two participants had more nuanced perspectives that incorporated characteristics of both the questioning and favouring attitudes. See Fig. 1.

### Exceptionalism vs normalisation and routinisation

#### Exceptionalism

Exceptionalism refers to the belief that HIV testing is different from other chronic disease testing [23, 24]. Accordingly, some participants stated that HIV testing was not as relevant, appropriate, or applicable as testing for or preventing other diseases. They saw it as being different from other conditions. One participant explained the clinical decision making process:

*What’s my idea with this patient? I’ve got to manage the diabetes. Try and get him to stop smoking and prevent cardiovascular disease. Should I test him for HIV? No, probably not.* (Participant 10, GP)

Exceptionalism was sometimes based on the participants’ belief that due to patient characteristics or socio-economic status, their patient population was not at risk of HIV. These HCPs felt they were able to profile their patients’ behaviour, which was underpinned by assumptions.

*In the last 12 months, the majority of my practice was one in which the population generally doesn’t have at-risk type behaviours--it’s an established upper middle class background so it’s a relatively low proportion*. (Participant 11, GP)

Other examples of HIV exceptionalism related to pre-test counselling and prevention education, which are not required for other common medical tests.

*To me the money would be better off increasing the awareness of risk factors rather than spending money on testing every single person. There’s no point in testing a nun, for example.* (Participant 21, NP)

Some participants stated that the recent increase in local HIV diagnoses indicated a need for more HIV prevention education, or a need for increased awareness, not a new testing protocol with abbreviated pre-test counselling. Similarly, condensing pre-test counselling would mean that an opportunity to educate people about HIV prevention could be missed.

*I know that there’s a high risk background for many of our clients, there’s needle sharing and risk-taking behaviour, but I don’t want [opt-out HIV testing] to replace education. The recent HIV statistics show that it’s the younger population or heterosexual population, people that are coming back from overseas…I think that’s where education has failed rather than [indicating a need to] have everybody tested. If you miss the dialogue then you miss an opportunity.* (Participant 1, GP)

Another aspect of HIV exceptionalism was the concept of stigma. Six participants who expressed discomfort with opt-out testing had beliefs about HIV that suggested they considered an HIV diagnosis a stigma. One participant indicated discomfort with the patient behaviours that led to HIV transmission:

*And you’d be surprised that plenty* [of patients] *admit to things that you just think, how could they?* (Participant 18, GP)

This discomfort with the sexual and drug use behaviours that could transmit HIV indicated that this participant would not routinely suggest HIV testing during patient consultations.

Specialist HCPs frequently mentioned GPs’ lack of up-to-date knowledge about HIV epidemiology, possibly related to its historic association with men who have sex with men, a stigmatised group.

*GP’s perceptions on the type of person that has HIV, which are inaccurate considering the large amount of heterosexual transmission in Australia, might not have caught up yet.* (Participant 22, GP)

This suggests that stigmatizing beliefs about HIV prevent GPs from considering testing people from other populations.

Another example of exceptionalism was the concern among some participants that opt-out HIV testing would cause excessive patient anxiety.

*I don’t think it’s a good idea to screen almost everyone like that. I don’t think that it would be very useful. It might trigger unnecessary anxiety and worries*. (Participant 16, NP)

Two participants mentioned potential for suicide as a possible patient response to an HIV-positive result, causing them to limit HIV testing to special appointments that allowed extra time for patient counselling.

*It could lead to suicides and so with the anxiety, you’re going to need longer appointments for somebody who’s coming for HIV testing.* (Participant 12, GP)

Any screening or diagnostic test can cause patient anxiety, but the participants’ degree of concern indicated that for them, HIV was unlike testing for other diseases and required special procedures.

### Normalisation and routinisation

Normalisation in HIV testing refers to offering testing in the same way for HIV as for as other medical tests for less stigmatised diseases. Despite the relatively low likelihood of finding an HIV-positive result, these participants believed that preventing late diagnosis was a benefit of the opt-out method.

*Lots of people are going to be negative and* [there would] *be just the odd positive one and you’d probably increase their life span by catching that person, so they’re not going to fall in a big heap in ICU with an AIDS-defining illness.* (Participant 7, GP)

Compared with HCPs who tended to question opt-out HIV testing, those favouring it tended to present HIV testing as a normal clinical activity, similar to testing for other chronic conditions (such as diabetes).

*I just don’t see HIV any different to any other long-term disease. Every time I test someone for blood sugar levels I don’t go on and on about how awful the disease diabetes is, you know?* (Participant 21, NP)

This participant stated that normalising HIV testing with the opt-out approach could decrease stigma, which could have broader societal benefits.

*I’m working in a sexual health clinic and essentially I offer it to everybody without taking regard of classic risk factors. We already have a slightly higher risk group and also I just don’t think there’s any reason not to test for HIV. We’re never going to get rid of the stigma around HIV unless we treat it like everything else.* (Participant 7)

Routinisation is similar to normalisation but refers to testing according to a standardised protocol or procedure, as opposed to testing according to clinical indication or risk factor [25]. Opt-out HIV testing would be akin to measuring adult patients’ blood pressure at every consult—a routine practice regardless of whether the patient has risk factors for high blood pressure or requests the test. In our study, participants who favoured opt-out HIV testing incorporated it into routine practice, rather than viewing it as an exceptional clinical event. As this participant indicates, making HIV testing a routine part of practice reassures patients that they are not being singled out.

*It makes it a routine and acceptable thing--the same as we would say, for every pregnant person we always check to make sure you don’t have chlamydia, to make sure your blood group, etc., and it normalises it to say there’s nothing good, bad or indifferent about being HIV positive. It just means it’s better to know than to not.* (Participant 24, specialist physician)

### Proof vs openness

#### Proof

Ten of the 11 participants who had a questioning attitude toward opt-out HIV testing cited a lack of rationale or evidence for a change in testing strategy. In their view, opt-out HIV testing would be reasonable only if there was risk factor-based evidence to support it.

*I think if there was an evidence base to say, this particular population have x number of risk factors, then* [an] *opt-out test should be offered, then I think that would be completely reasonable.* (Participant 11, GP)

Some referred to evidence-based practice concepts such as statistical significance, pre-test probability, and survival rates, like this participant:

*I would question the premise of offering HIV testing to everyone. If we’re going to offer a group of people a battery of tests, there has to be good evidence that we’re going to find that disease in that population. If you can prove that you’re going to pick up new infections at a statistically significant rate and prevent adverse outcomes and improve treatment and survival rates, then it’s a reasonable thing to do. (*Participant 14, GP)

Some comments indicated a lack of knowledge about the high sensitivity and specificity of current HIV tests, overestimating the risk of a false positive result, such as this GP:

*I personally would refuse to be tested because I don’t want to deal with false positives--it will have some anxiety related to it until you get the results back.* (Participant 18, GP)

This participant did not mention the demonstrated benefits of early HIV diagnosis, and seemed skeptical that knowing one’s HIV status could be a positive effect of increased testing.

*I’d like to see the evidence first* [about] *the benefit, that every single person in the country knows their HIV status. If we tested every single person for HIV, I’d like to know how many more people we are picking up that we’re missing. I’d like to know if early diagnosis reduces complications afterwards. (*Participant 21, NP)

Need for proof was often framed in terms of cost-effectiveness. Participants stated that if HIV testing were increased, more beneficial health interventions could be neglected.

*You’d need to look at whether or not the number of new diagnoses you made is worth the cost or whether your portion of health money could go into something else that would be potentially more valuable.* (Participant 3, GP)

Need for proof was also associated with a fear of reprimand. Participants indicated that if they performed more HIV tests, Australian government funding bodies (Medicare) would question its appropriateness.

*Medicare would be tapping on my door asking why I’m doing so many HIV tests, and can I justify the expense to the government?* (Participant 24, GP)

The need for proof of concept was seen as important to protect HCPs delivering what they perceived to be potentially contentious care.

### Openness to new approaches

Openness to new testing approaches was a common attitude among participants who favoured opt-out HIV testing. Participants often referred to the benefits of increased HIV testing and mentioned positive outcomes from other countries. One participant stated that opt-out testing could prompt HCPs to test more patients for HIV.

*So, I think it’s a good thing to have and it just makes us think about us doing it more as opposed to thinking about doing it less.* (Participant 6, NP)

This participant was aware of evidence about opt-out HIV testing from other countries and considered its implications for her practice:

*I think that it’s being shown in countries like England around the pregnancy testing and I think it is dangerous when people do their own risk assessment. And what they found in England is that obstetricians got it wrong.* (Participant 23, GP)

Unlike the HCPs who had a more questioning attitude toward opt-out HIV testing, participants who were open to the approach thought that the cost of opt-out HIV testing was reasonable and would be acceptable to the public.

*To me it seems a well-run, well-managed process…it’s not a massive cost on the public purse that people get agitated about.* (Participant 8, GP)

This participant advocated expanding opt-out testing to the emergency department to expand access to HIV testing:

*You could do it in EDs, you could do it in other settings where there’s a much bigger throughput.* [ED] *might not be a bad spot to target because they’re seeing a cross sections of people that wouldn’t usually access health services.* (Participant 7, GP)

## Discussion

This initial study of Australian HCPs’ views on opt-out HIV testing revealed new insights and some surprise findings. The majority of participants’ views fell into one of two mutually exclusive categories: favouring or questioning. Participants who tended to question opt-out HIV testing doubted its relevance to Australia, focused on the method’s flaws, and emphasised potentially negative consequences. Surprisingly, they did not identify some of the barriers to opt-out HIV testing that commonly appeared in previous research, such as operational issues [15, 26, 27], time constraints [28–30] or inadequate linkage to HIV care [31]. Conversely, participants who were generally comfortable with opt-out HIV testing had similar views to those identified in previous research, such as placing HIV testing in the same domain as testing for other diseases [27, 31]. Finally, pilot studies provide useful data about changes in clinical practice before wider implementation, but participants in this study did not suggest a pilot test of opt-out HIV testing in an Australian setting.

Participants who had a questioning attitude toward opt-out HIV testing doubted its relevance to the general Australian population, citing a lack of evidence for efficacy in lower prevalence populations. Australia has a low HIV prevalence by global standards; however, the idea that HIV testing is irrelevant in areas with low rates of infection has been considered the “false security” of low prevalence [29] (p.75), potentially resulting in delayed HIV diagnosis. Participants in this category stated they were able to accurately determine which patients should be tested based on their characteristics (such as age or suburb of residence), a strategy that previous studies have shown to be ineffective in identifying HIV infections [5, 32–34]. Opt-out HIV testing could decrease the potential for error in patient risk assessment.

Participants who questioned the opt-out approach to HIV testing tended to focus on its potential flaws, which may reflect HCPs’ analytical style [35–39]. While HCPs are trained to look for logical negatives (for instance, they might ask themselves, “what is wrong with this picture?” in the diagnostic process), this frame of mind could lead to pessimism about changes in practice [40]. Studies have shown that HCPs are particularly reluctant to change their practice in view of new evidence [35] and are prone to “paralysis by analysis”—which occurs when the discussion about a change becomes so arduous that no action is taken [41]. Our data also suggest that HCPs sometimes make decisions about HIV testing based on personal beliefs and values, not necessarily logical reasoning, which is consistent with behavioural economic theory [42]. A change in HIV testing practice should take into account these common HCP analytical patterns. A small, incremental trial should provide feasibility data that could be used to determine the efficacy of opt-out HIV testing, without ‘forcing’ HCPs into a major change too quickly [43].

Some HCPs seemed to need more support and education about HIV testing and disclosure of results. Participants who viewed opt-out HIV testing less favourably were worried about potential negative consequences, such as stigma, anxiety and suicide, supporting previous research findings [29, 31, 44, 45]. Studies have shown that HCP education can facilitate implementation of opt-out HIV testing (which would be especially important in Australia, given that many participants in this study had limited knowledge of, or experience with, the approach) [46]. Academic detailing (brief, one-on-one education sessions) prior to large-scale implementation of opt-out HIV testing has been shown to increase its acceptance among HCPs [47]. Peer-based education could be particularly effective because it provides a social reference for HCPs (who are often unconsciously influenced by their peers) [48, 49]. Because many participants in our study were worried about disclosing HIV-positive results, HCPs should receive education about best practices for disclosure, ideally with a protocol for linkage to HIV care already in place [14, 45, 50]. Education programs have also been shown to promote positive HCP attitudes toward opt-out HIV testing, with HCPs citing patient behaviour change and reduced HIV transmission as affirming aspects of the approach [31, 51].

Although they tended to disapprove of opt-out HIV testing for the general Australian population, participants who had a questioning attitude had flexible beliefs: they were not necessarily opposed to opt-out HIV testing, provided there was Australian evidence of efficacy and cost-effectiveness. They were willing to revise their attitudes based on new information or experience, which has been associated with increased acceptance of opt-out HIV testing [32]. Research about opt-out HIV testing has shown that ongoing quality improvement activities (particularly with HCP participation) are essential for making changes in practice [52]. Similarly, knowledge translation studies have demonstrated the importance of short-term, small-scale pilot tests before expanding new programs [53]. Surprisingly, regardless of whether they were comfortable with opt-out HIV testing, participants in this study did not suggest short-term trials or small-scale quality improvement projects before considering broader implementation.

Few participants who were comfortable with opt-out HIV testing mentioned its cost, indicating that for them, potential benefits of the approach outweighed the costs. There is scant international evidence about HCP views on the cost of opt-out HIV testing. Further, most studies on opt-out HIV testing were conducted in the US and funded by federal grants, which may account for the lack of cost concerns among US participants [53]. Future research should explore HCP attitudes about the cost of opt-out HIV testing and their effects on changes in testing practice.

This study has provided an initial insight into the acceptability of opt-out HIV testing in Australia. While some HCPs have embraced the opt-out approach, risk factor-based HIV testing remained entrenched among some participants. Subsequent research could best inform future HIV testing recommendations by addressing the issues raised by the HCPs who viewed opt-out HIV testing less positively [54]. Given some participants’ concerns about over-testing and excessive cost, existing Australian cost-effectiveness modelling data should be more widely disseminated and replicated [55]. Efficacy and feasibility data about opt-out HIV testing through a pilot study in an Australian context could meet HCPs’ need for evidence [32]. Finally, Australian research on opt-out HIV testing should explore the operational barriers (such as time constraints) identified in international research.

### Methodological considerations

Our results should be interpreted within their methodological context. The interviews were conducted by a clinician-researcher (SL) who had experience with opt-out HIV testing in the US (where the opt-out approach has been recommended for nearly a decade). During the interviews, the participants sometimes asked the researcher questions about opt-out HIV testing in the US, which was a diversion from the aim of the research, and may have influenced their views (but also supported their need for education). Although we actively recruited participants with negative or differing opinions about opt-out HIV testing, the sampling method may have resulted in a limited spectrum of perspectives. Due to the small sample size, we were not able to make separate subgroup analyses within the sample, such as a comparison of attitudes between HCPs with different levels of experience. Another limitation was that the participants practiced in one Australian state, which may not represent the full range of Australian HCPs’ views.

## Conclusion

Most HCPs in this study had dichotomous attitudes toward opt-out HIV testing, reflecting contrasting analytical styles. While some HCPs viewed it favourably, with the perceived benefits outweighing the perceived costs, others preferred to have evidence of efficacy and cost-effectiveness. In response to the findings from this study, we have designed a pilot test of opt-out HIV testing in an Australian general practice. The pilot test will explore HCP and patient experiences with the opt-out approach, compare the number of HIV tests and results between opt-out and traditional testing, and analyse the cost impact of opt-out testing. Findings from this pilot test should help to inform the desirability of introducing opt-out HIV testing in Australia.

## Additional file

Additional file 1:
**Interview guide.** (DOCX 16 kb)
