# Supplementary material for: Australian health care providers’ views on opt-out HIV testing
Source: BMC Public Health. 2015 Sep 14;15:888. doi: 10.1186/s12889-015-2229-9 (PMC4570459; doi:10.1186/s12889-015-2229-9)
Supplement: Additional file 1: — Interview guide. (DOCX 16 kb) [file 12889_2015_2229_MOESM1_ESM.docx]

**
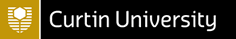
Interview Guide**

Interviewer will provide a definition of opt-out HIV testing after question one.

1. Tell me about the process you go through when you consider HIV testing (such as risk factors, clinical presentation, possible diagnoses, client acceptability).

*Opt-out testing: “Opt-out’’ HIV testing is defined as conducting HIV testing after notifying patients that the test will be conducted and that they may decline or defer testing.*

1. Tell me about your thoughts on opt-out testing—what benefits do you think there would be for clients? For HCPs?
2. What barriers do you think there would be for clients? For HCPs?
3. Do you have any suggestions for how opt-out HIV testing could be made easier for HCPs?
4. What ethical issues would you consider if you were to perform opt-out HIV testing?
